# Editorial: Ischemic Myocardial Injury and Protection: From Bench to Bedside

**DOI:** 10.3389/fcvm.2022.940298

**Published:** 2022-05-24

**Authors:** Hui Gong, Hongmei Tan, Yaoliang Tang

**Affiliations:** ^1^Shanghai Institute of Cardiovascular Diseases, Zhongshan Hospital, Fudan University, Shanghai, China; ^2^Department of Pathophysiology, Zhongshan School of Medicine, Sun Yat-sen University, Guangzhou, China; ^3^Department of Medicine, Medical College of Georgia at Augusta University, Augusta, GA, United States

**Keywords:** ischemic heart disease (IHD), myocardial injury, protection, metabolic syndrome (MS), heart failure

Ischemic heart disease (IHD) remains the primary cause of global mortality (about 17% of all death), and has been considered as a life-threatening problem (1). Myocardial ischemia is usually caused by atherosclerotic plaque complication or microvascular dysfunction, which induces cardiomyocytes loss, apoptosis, autophagy, inflammation or fibrosis, resulting in cardiac dysfunction or heart failure (2–4). Although currently basic, translational, and clinical findings have provided a massive amount of information about ischemic myocardial injury and protection, acumulating evidence suggests a complex pathophysiology of IHD. To better understand this complicated disease and provide future perspectives, we have the pleasure of bringing together the Research Topic “*Ischemic Myocardial Injury and Protection: From Bench to Bedside*” for the readers of *Frontiers in Cardiovascular Medicine*. This Research Topic includes twelves article and one review gathering an interdisciplinary overview of molecular mechanism, high risk factor, prognosis, complication, and novel treatment of ischemic myocardial injury.

The high mortality of IHD is attributed to sudden cardiac death (SCD), which results from lethal ventricular arrhythmias of ventricular tachycardia (VT) (5). Qi et al. found blockade of Na_V_1.8 in cardiac ganglionated plexi (GP) increased the incidence of ventricular arrhythmias in acute myocardial infarction (AMI) hearts. Present results indicated suppression of GP activity might enhance the genesis of ventricular arrhythmia; Na_V_1.8 plays an important role in cardiac conduction *via* regulation of action potential firing in intracardiac neurons. Qi et al. observed that Na_V_1.8 was expressed in canine GPs, blockade of Na_V_1.8 with A-803467 shortened ventricular effective refractory period (VERP), ventricular 90% of action potential duration (APD_90_), and decreased ventricular fibrillation threshold (VFT) during AMI, and also reduced heart rate in response to GP stimulation. Although the detailed mechanisms are needed to further study to clarify, the study suggests Na_V_1.8 may be as a potential novel therapeutic target for anti-arrhythmic intervention and prevention of SCD of patients with IHD.

Exosomes derived from a variety of cardiac cells have been reported to regulate intercellular communication and crosstalk in ischemic heart by the transfer of miRNA or other proteins (6). Li, Zhao et al. demonstrated plasma exosomes at the late phase of remote ischemic pre-conditioning (RIPC) alleviated myocardial ischemia-reperfusion injury *via* exosomal miR-126a-3p. On the basis of cardiac-protection of exosomes at the late phase of RIPC, they found the significant enrichment of miR-126a-3p in RIPC-exosome by miRNA array. Mechanistically, RIPC-exosomal miR-126a-3p, activated reperfusion injury salvage kinase (RISK) through Akt and Erk1/2 to inhibit apoptosis. This study is interesting, and presents a critical mechanism underlying the exosomal miR-126a-3p might be a novel cardioprotective molecule in the prevention and rehabilitation of ischemic myocardial injury. However, Tu et al. showed that sepsis-exosomes promoted the pyroptosis of cardiomyocytes and worsened cardiac dysfunction through miR-885-5p *via* HMBOX1. These findings suggest the different mechanisms or functions of exosomes in ischemic myocardial injury may dependent on exosomal source of pathophysiological process or cell-types.

Circular RNAs (circRNAs), function as a sponge and bind specific miRNAs to exert critical effects in ischemic heart diseases and vascular diseases. Liu et al. verified that downregulation of rno_circRNA_0009197, and upregulation of rno_circRNA_0005304, rno_circRNA_0005506, rno_circRNA_0005818, and rno_circRNA_0009301 in hypertensive aorta. Three promising circRNA-miRNA-mRNA regulatory axes, including rno_circRNA_0005818/miR-615/NOTCH1, rno_circRNA_0005818/miR-10b-5p/STAT3, and rrno_circRNA_0009197/miR-509-5p/FOXO3, may play potential roles in hypertensive vascular remodeling and dysfunction. The findings indicate that circRNAs are the vital therapeutic targets for ischemic heart diseases and vascular diseases.

Hydrogen sulfide (H_2_S), an endogenously generated gaseous transmitter, has been shown to be cardiac protective in ischemic myocardial injury or heart failure (7, 8). Li, Xie et al. explored the cardiac protection and potential mechanism of S-propargyl-cysteine (SPRC), a novel modulator of endogenous H_2_S. SPRC has been identified to be protective in myocardial infarction *via* a H2S-related pathway (9). In Li, Xie et al. study, SPRC treatment alleviated cardiac systolic dysfunction and myocardial hypertrophy or fibrosis, accompanied by a reduction in myocardial lipid accumulation in *db/db* diabetic mice. The functional improvement by SPRC was associated with activation of insulin receptor signaling and subsequent enhancement of glucose uptake in cardiomyocyte. These data suggest that SPRC might be a promising medication for patients with IHD and/or diabetic cardiomyopathy.

Clinical studies has revealed more risk factors including metabolic syndrome or metabolism disorder for coronary artery disease (CAD) or IHD. Metabolic syndrome (MS) is characterized by a cluster of risk factors including central obesity hypertension, hyperglycemia, and dyslipidemia, significantly increases the risk for cardiovascular disease (CVD), or the morbidity and mortality of CVD patients (10). Zhao F. et al. conducted this cross-sectional study with 1,958 participants from the Northern Shanghai Study aged over 65 years without a history of CVD. The authors' data revealed that a positive association between MS and various asymptomatic cardiovascular injury in an elderly Chinese population. Importantly, MS was significantly associated with left ventricular hypertrophy (LVH), LV diastolic dysfunction, arteriosclerosis, and microalbuminuria. Yu et al. performed a single-center, observational cohort study enrolled 183 acute heart failure (AHF) patients and evaluate the association between the serum circulating free fatty acids (FFAs) level and all-cause mortality or HF rehospitalization. Serum FFAs levels were positively correlated to the risk of death or HF rehospitalization, which was not related to the status of insulin resistance. In this retrospective cross-sectional study, patients who underwent elective percutaneous coronary intervention (PCI). Li, Li et al. found elevated HbA1c increased the atherosclerotic plaque vulnerability as evidenced by the thinner minimal fibrous cap thickness (FCT), higher lipid index, and greater macrophage index. Moreover, elevated average follow-up HbA1c level was positively associated with higher visit-to-visit variability of lipids levels, including LDL-C, HDL-C, non-HDL-C, TC, and TG. Our basic research showed deficiency of cardiac LRP6 induced lipid accumulation and heart failure, and overexpression of low-density lipoprotein receptor-related protein 6 (LRP6) protects heart from ischemic-reperfusion injury in mice (11–13). These data suggested that interventions for metabolic disorders may be critical for improvement of prognosis of CVD.

In treatment of CAD or IHD, PCI has become one of most effective treatments for patients with AMI. But it also induces some complications in patients such as contrast induced nephropathy (CIN), caused by intravascular contrast media. Mo et al. identified three novel predictors for CIN, baseline uric acid level, creatine kinase-MB level, and log (N-terminal pro-brain natriuretic peptide) level, in patients with CAD and relatively normal renal function (NRF). The authors developed the simplified risk score for contrast induced acute kidney injury (CI-AKI) model on the basis of the new criteria. It exhibits accurate predictive performance to identify high-risk patients with CIN. For drug treatment, β-blockers predominantly increase sympathetic nerve activity, and become the first-line agent for cardiovascular diseases. However, Dai et al. found β-blockers adversely affected erectile dysfunction (ED) improvement conferred by coronary revascularization in patients with early onset of coronary artery disease (EOCAD). This study calls for re-considering β-blockers as the first-line option for the EOCAD patients with ED.

Some researchers explored novel intervention for ischemic myocardial injury. Electrical stimulation (ES), a non-invasive and safe physiotherapy, is considered to be a promising clinical application in the treatment of IHD, which attracts more and more attention. Zhao Y. et al. systemically reviewed the types, the beneficial effects and molecular mechanisms of ES in the treatment of IHD. ES promotes angiogenesis by stimulating VEGF or FGF2 secretion, reduces cardiomyocytes apoptosis by inhibiting the production of TNF-α and intercellular adhesion factor-1 (ICAM-1), inhibits cardiac hypertrophy by suppression of ERK1/2 signaling, in the ischemic myocardium. ES also protects heart from ischemic injury by regulation of autophagy, inflammatory response and the production of ROS. This exhaustive review prompts to investigate in depth the molecular mechanisms underlying ES to develop new therapeutic opportunities. The authors suggest that appropriate parameters and implementation methods and locations of ES are needed further study to provide better scientific data for clinical application in IHD. Moreover, cardiac shock wave therapy (CSWT), has been reported to be an effective and non-invasive therapy, which is mainly applied to ameliorate left ventricular remodeling after (AMI). Wang et al. found the application of CSWT significantly improved cardiac function and myocardial fibrosis by activation of PI3k /AKT signaling in experimental AMI model. Moreover, renal denervation (RDN) reduces the activation of the sympathetic nervous system (SNS) and renin-angiotensin-aldosterone system (RAAS) for hypertension treatment. Chen et al. investigated the cardioprotective effects of immediate renal denervation (RDN) after AMI and explored the underlying mechanism. They found immediate RDN could improve cardiac remodeling and function after AMI *via* regulation of IL-33/ST2 and downstream signaling. The study suggests RDN may become another viable application in emergency PCI beyond hypertension.

In conclusion, the articles collected for this Research Topic present current progress and perspectives on the pre-clinical, clinical and epidemiological studies on the ischemic myocardial injury and protection. We believe that these articles advance our understanding and perspectives on cardiovascular diseases fields. We are grateful for all contributors for sharing their interesting work for the Research Topic.

## Author Contributions

HG wrote the manuscript. HT and YT revised the manuscript. All authors listed have made a substantial, direct, and intellectual contribution to the work and approved it for publication.

## Funding

This work was supported by grants from National Natural Science Foundation of China (NSFC) (No. 82070232 for HG, Nos. 82170357 and 81873514 for HT) and Science and Technology Commission of Shanghai Municipality (21XD1403300 for HG).

## Conflict of Interest

The authors declare that the research was conducted in the absence of any commercial or financial relationships that could be construed as a potential conflict of interest.

## Publisher's Note

All claims expressed in this article are solely those of the authors and do not necessarily represent those of their affiliated organizations, or those of the publisher, the editors and the reviewers. Any product that may be evaluated in this article, or claim that may be made by its manufacturer, is not guaranteed or endorsed by the publisher.
